# The transcriptional regulators GATA6 and TET1 regulate the TGF-β pathway in cancer-associated fibroblasts to promote breast cancer progression

**DOI:** 10.1038/s41420-025-02438-4

**Published:** 2025-04-11

**Authors:** Mohammad H. Ghazimoradi, Sadegh Babashah

**Affiliations:** Department of Molecular Genetics, Faculty of Biological Sciences, Tarbiat Modares University, Tehran, Iran

**Keywords:** Cancer microenvironment, Mechanisms of disease, Breast cancer

## Abstract

Cancer-associated fibroblasts (CAFs) are pivotal drivers of tumor progression, yet the molecular mechanisms underlying their activation remain incompletely understood. Here, we identified the TET1/SMAD4/GATA6 regulatory axis as a central mechanism governing CAF transformation and function in breast cancer. Through integrative in vitro and in vivo models, we demonstrated that TET1, an epigenetic modulator, demethylates the *SMAD4* promoter, enhancing SMAD4 expression. SMAD4 transcriptionally upregulates *GATA6*, which amplifies TGF-β signaling by directly activating the *TGF-β* promoter, establishing a self-reinforcing feedforward loop critical for CAF identity and stromal-tumor crosstalk. GATA6 and TET1 were significantly upregulated in breast CAFs compared to normal fibroblasts (NFs) and TGF-β-induced CAFs. Loss- or gain-of-function experiments revealed that these regulators control CAF survival, marker expression, and secretion of pro-tumorigenic factors. Knockdown of *GATA6* or *TET1* reduced CAF-mediated migration and invasion of breast cancer cells in vitro, while their overexpression enhanced cancer cell aggressiveness. Mechanistically, TET1-mediated epigenetic remodeling and GATA6-driven transcriptional activation converge on the TGF-β/SMAD pathway, sustaining CAF activation. In vivo, tumors derived from GATA6- or TET1-depleted CAFs exhibited reduced growth, proliferation, and CAF engraftment, underscoring their role in tumor progression. These findings position GATA6 and TET1 as promising targets to disrupt CAF-driven tumorigenesis, offering novel strategies for breast cancer treatment. By unraveling the epigenetic-transcriptional interplay within the tumor microenvironment, this study advances our understanding of stromal reprogramming and its implications for precision oncology.

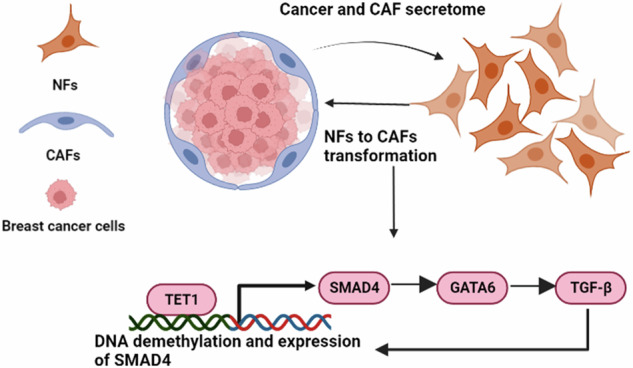

## Introduction

Cancer-associated fibroblasts (CAFs) are key components of the tumor microenvironment (TME). They are defined as fibroblasts within and surrounding tumor tissues that originate from activated normal resident fibroblasts (NFs) or transdifferentiate from non-fibroblastic lineages [1, 2]. The activation of NFs into CAFs is primarily induced by tumor-derived factors such as transforming growth factor-beta (TGF-β) secreted by cancer cells, which drive CAFs to support tumor malignancy [3, 4]. These heterogeneous CAF populations can be classified into distinct subtypes that promote angiogenesis, tumor progression, and drug resistance [5, 6]. While the functional significance of CAFs in cancer is well-documented, the molecular mechanisms underlying their activation remain incompletely understood [7]. For instance, cytokines such as TGF-β, osteopontin, and interleukin (IL)-1—produced by cancer cells or immune cells—activate signaling pathways that trigger the conversion of stromal fibroblasts into CAFs [8]. Among these, the TGF-β/SMAD pathway is considered central [9]. Emerging evidence suggests that transcription factors may also play critical roles in fibroblast transformation, potentially acting in concert with canonical pathways. Yes-associated protein 1 (YAP1), a transcriptional coactivator in normal fibroblasts, regulates SRC transcription by forming a protein complex with TEA domain transcription factor-1. This interaction leads to the activation of cytoskeletal proteins and, ultimately, the transformation of fibroblasts into CAFs [10]. Downregulation of Caveolin-1 or mitochondrial transfer from cancer cells can also be used to metabolically reprogram fibroblasts [11]. TWIST1 has emerged as a promising gene for reprogramming fibroblasts into CAFs, although the underlying mechanisms are yet to be fully understood [12]. In addition to transcription factors, epigenetic regulatory enzymes play a critical role in reprogramming by modulating upstream and downstream pathways [13, 14]. In this context, identifying the appropriate molecular pathways could provide a strategy to inhibit the transformation of normal fibroblasts (NFs) into CAFs [15].

The only GATA family member expressed in the distal epithelium of the embryonic lung is GATA-binding factor 6 (GATA6), a zinc-finger transcription factor essential for visceral endoderm and heart development, as well as for myofibroblast function [16, 17]. Despite the well-established role of GATA6 in development, its function in cancer remains unclear. In gastric cancer, GATA6 inhibits migration and metastasis by modulating the miR-520b/CREB1 axis [18]. It has a suppressive role in lung cancer [19]. In contrast, GATA6 may contribute to the development and progression of oral malignancies [20]. Additionally, GATA6 is overexpressed in triple-negative breast cancer, where it upregulates slug expression, promoting epithelial-to-mesenchymal transition (EMT) in breast cancer cells [20]. In idiopathic pulmonary fibrosis, GATA6 is expressed in quiescent myofibroblasts [21]. This protein also modulates tracheal fibrosis by activating fibroblast to myofibroblasts through the WNT pathway [22]. Ten-eleven translocation methylcytosine dioxygenase 1 (TET1) is a member of the TET family of enzymes. By demethylating various genes, TET1 regulates their expression and, consequently, their associated signaling pathways [23, 24]. It has been shown that TET1 can influence cancer progression by demethylating key pathways, such as the WNT pathway [25]. Additionally, TET1 plays a crucial role in facilitating and establishing cell reprogramming; its silencing can disrupt the differentiation process [26, 27]. Notably, TET1 also aids in the programming of induced pluripotent stem cells [28, 29].

Although GATA6 and TET1 are implicated in diverse cellular processes, their specific functions in CAFs remain poorly characterized. This study aimed to elucidate the molecular mechanisms through which the TET1/SMAD4/GATA6 regulatory axis governs CAF activation in breast cancer, focusing on its role in sustaining TGF-β signaling, promoting stromal-tumor crosstalk, and driving tumor progression. The broader objective was to identify novel therapeutic targets capable of disrupting CAF-mediated oncogenesis.

## Results

### GATA6 and TET1 are upregulated in breast cancer-associated fibroblasts

**Fig. 1:**
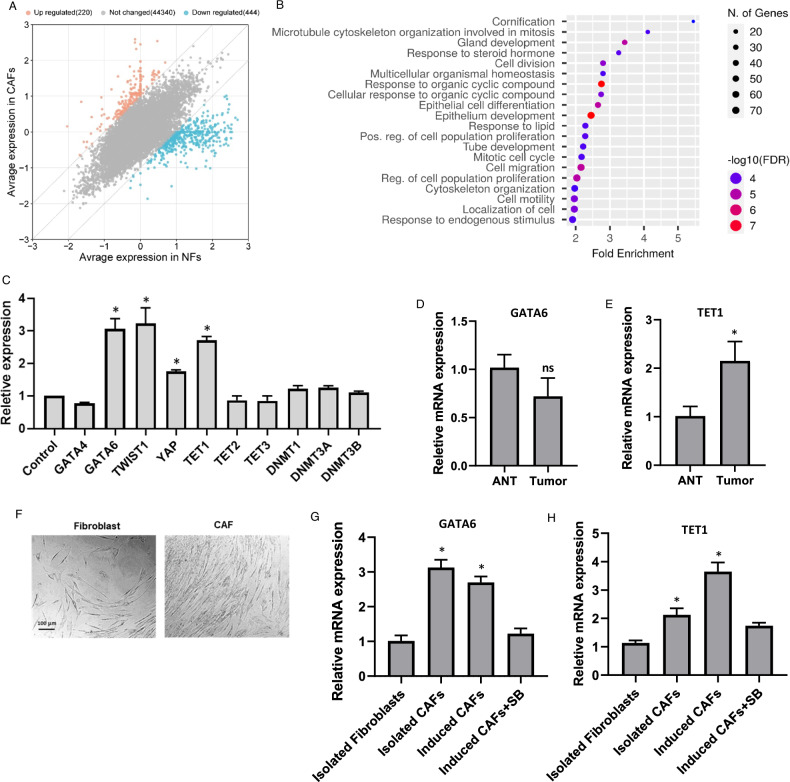
In silico and expression analyses identify GATA6 and TET1 as key regulators in breast cancer-associated fibroblasts. **A** Differentially expressed genes (DEGs) in CAFs versus normal fibroblast (NFs). Data were obtained from the GEO dataset E-GEOD-29270 (n = 58; CAFs = 35, NFs = 23). The red dots represent upregulated DEGs, and the blue dots represent downregulated DEGs. **B** Pathway analysis of DEGs in CAFs versus NFs. **C** Relative mRNA expression of candidate genes in CAFs compared to NFs (Control). Data were obtained from GEO dataset E-GEOD-29270 (*n* = 58; CAFs = 35, NFs = 23). (*P < 0.05). **D** Relative mRNA expression of GATA6 in breast tumors compared to adjacent non-tumor breast tissues, as measured by qRT-PCR (*n* = 10 for each group). No significant difference was observed (*P* > 0.05). **E** Relative mRNA expression of GATA6 in breast tumors compared to adjacent non-tumor breast tissues, as measured by qRT-PCR (*n* = 10 for each group) (*P < 0.05). **F** Representative images of isolated fibroblasts and CAFs from breast cancer tumors. Scale bar: 100 µm. **G** Relative mRNA expression levels of GATA6 in NFs, CAFs, TGF-β-induced CAFs, and TGF-β-induced CAFs treated with the TGF-β/SMAD inhibitor SB 431542 (SB), as measured by qRT-PCR (*P < 0.05). **H** Relative mRNA expression levels of TET1 in NFs, CAFs, TGF-β-induced CAFs, and TGF-β-induced CAFs treated with the TGF-β/SMAD inhibitor SB 431542 (SB), as measured by qRT-PCR (*P < 0.05). Error bars represent standard deviation (SD). All statistical tests had a post hoc power >80%. ANT adjacent non-tumor breast tissues.

Analysis of a published transcriptomic dataset comparing normal fibroblasts (NFs) and cancer-associated fibroblasts (CAFs) in breast tissues identified differentially expressed genes (DEGs) enriched in CAFs (Fig. 1A). Gene set enrichment analysis (GSEA) revealed that these DEGs were predominantly DNA-binding proteins associated with epithelial development, cytoskeletal remodeling, and proliferative pathways (Fig. 1B). To investigate drivers of this phenotype, we focused on transcription factors implicated in epithelial differentiation or stromal reprogramming. Among candidates, GATA6 and GATA4 emerged as potential regulators due to their roles in epithelial-stromal crosstalk. Strikingly, GATA6 expression was significantly upregulated in CAFs compared to NFs (*P* < 0.05; Fig. 1C), while GATA4 showed no differential expression. To validate our findings, we assessed YAP1 and TWIST1, known mediators of stromal activation. Consistent with prior studies, both genes were significantly upregulated in CAFs compared to NFs (*p* < 0.05; Fig. 1C), confirming the robustness of our dataset. Given the enrichment of CxxC domain-containing proteins among differentially expressed genes in CAFs (*P* value = 5.10E-03), we hypothesized a role for epigenetic regulators in fibroblast reprogramming. Strikingly, TET1 expression was markedly elevated in CAFs (*p* < 0.001 *vs*. NFs; Fig. 1C), whereas other TET family members (TET2/3) and DNA methyltransferases (DNMTs) showed no significant differential expression. Of clinical relevance, we first quantified the expression of GATA6 and TET1 in breast tumors and adjacent normal breast tissues. While GATA6 showed no significant differences (*P* > 0.05), TET1 expression was significantly elevated in tumor tissues compared to adjacent non-tumor breast tissues (*P* < 0.05; Fig. 1D, E). As previously demonstrated, TGF-β treatment drives the conversion of normal fibroblasts (NFs) into CAF-like cells in vitro [30, 31]. In this study, TGF-β-induced CAFs were used exclusively for comparative analysis, while isolated primary CAFs and normal fibroblasts (Fig. 1F) were employed for functional assays. Although qRT-PCR revealed no significant difference in GATA6 expression between breast tumor tissues and adjacent non-tumor breast tissues (Fig. 1D), GATA6 was markedly upregulated in both isolated and TGF-β-induced CAFs compared to NFs (*P* < 0.05; Fig. 1G). Strikingly, this upregulation was abolished by co-treatment with the TGF-β/SMAD inhibitor SB 431542 (SB), confirming TGF-β’s role in GATA6 induction (*P* < 0.05; Fig. 1G). Similarly, TET1 transcript levels were highest in TGF-β-induced CAFs, followed by isolated CAFs, with minimal expression in NFs and SB-treated TGF-β-induced CAFs (*P* < 0.05; Fig. 1H). These findings implicate TGF-β-driven GATA6 and TET1 upregulation as hallmarks of CAF activation.

### GATA6 and TET1 regulate the identity, survival, and function of breast cancer-associated fibroblasts

**Fig. 2:**
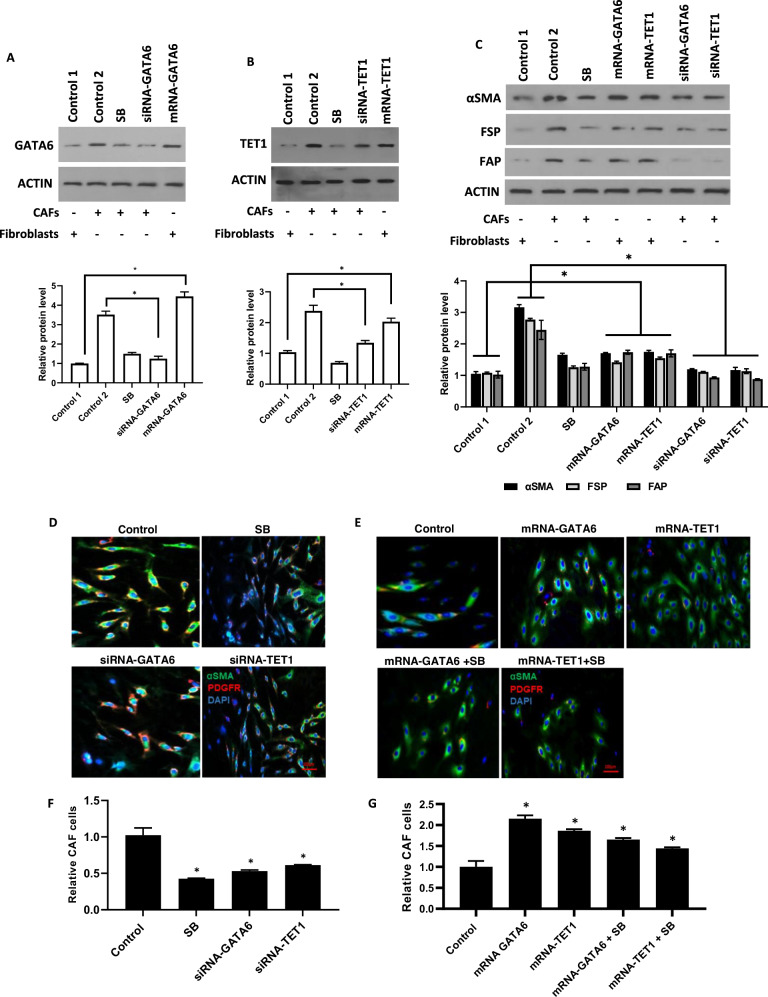
GATA6 and TET1 regulate CAF identity and abundance. **A** Western blot analysis of GATA6 protein levels in fibroblasts and CAFs treated with siRNA (loss-of-function) or mRNA (gain-of-function) versus corresponding controls. Control 1: NFs transfected with FLuc RNA; Control 2: CAFs transfected with scramble siRNA. ACTIN served as a loading control. The cropped blots are presented correspondingly. Full-length blots are presented in Supplementary Fig. 1. **B** Western blot analysis of TET1 protein levels in fibroblasts and CAFs treated with siRNA or mRNA versus controls (as in **A**). ACTIN served as a loading control. The cropped blots are presented correspondingly. Full-length blots are presented in Supplementary Fig. 1. **C** Protein levels of CAF markers (αSMA, FAP, FSP-1) after knockdown or overexpression of GATA6 and TET1. Representative blots from three independent experiments. ACTIN served as a loading control. The cropped blots are presented correspondingly. Full-length blots are presented in Supplementary Fig. 2. For the quantitative analysis of protein levels, band density was assessed using ImageJ software and represented as relative intensity. *P < 0.05 (**A**–**C**). **D**. Immunofluorescence staining showing the percentage of αSMA^+^/PDGFRβ^+^ cells in CAFs treated with siRNA-GATA6 or siRNA-TET1, compared to scramble siRNA-transfected CAFs (Control). Scale bar: 100 µm. (*P < 0.05). Error bars represent standard deviation (SD). **E** Immunofluorescence staining showing the percentage of αSMA^+^/PDGFRβ^+^ cells in fibroblasts transfected with mRNA-GATA6 or mRNA-TET1, either alone or in combination with SB 431542 treatment, compared to FLuc mRNA-transfected fibroblasts (Control). Scale bar: 100 µm. *P < 0.05. Error bars represent standard deviation (SD). **F** siRNA-mediated knockdown of GATA6 and TET1 in CAFs reduced their population relative to scrambled siRNA-treated CAFs (Control). **G** Overexpression of GATA6 or TET1 via mRNA transfection in normal breast fibroblasts (NFs) increased the number of CAF-like cells compared to FLuc-transfected NFs (Control).

**Fig. 3:**
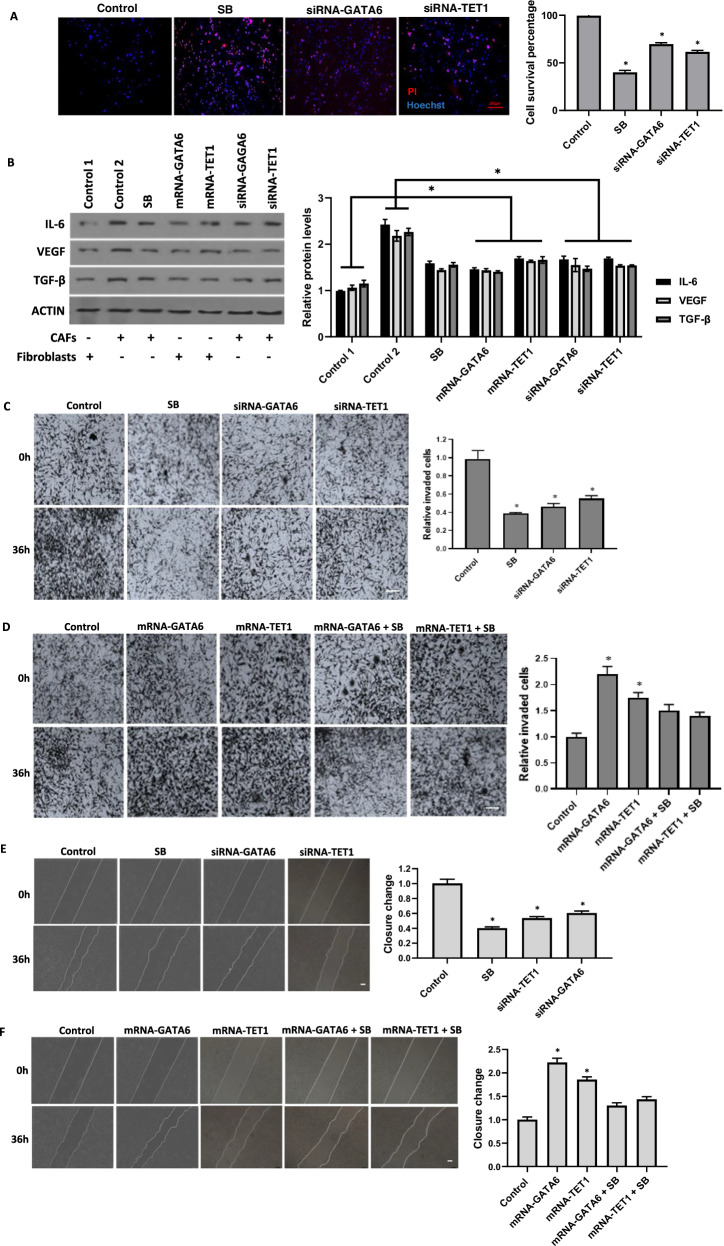
GATA6 and TET1 regulate CAF proliferation and their influence on MDA-MB-231 breast cancer cell migration and invasion. **A**. Hoechst 33342/propidium iodide (PI) double staining showing the inhibitory effect of GATA6 and TET1 knockdown on CAF survival compared to scramble siRNA-transfected CAFs (Control). Quantitative assessment revealed that CAFs transfected with siRNAs targeting GATA6 and TET1, or treated with SB 431542, showed a significant reduction in survival (*P < 0.05). **B** Protein levels of IL-6, VEGF, and TGF-β in normal breast fibroblasts (NFs) transfected with GATA6 mRNA or TET1 mRNA and CAFs transfected with siRNA targeting GATA6 (siRNA-GATA6) or TET1 (siRNA-TET1), compared to respective controls (NFs: FLuc-transfected [Control 1]; CAFs: scrambled siRNA-transfected [Control 2]). ACTIN served as a loading control. Representative blots from three independent experiments. ACTIN served as a loading control. The cropped blots are presented correspondingly. Full-length blots are presented in Supplementary Fig. 3. For the quantitative analysis of protein levels, band density was assessed using ImageJ software and represented as relative intensity. *P < 0.05. **C** Transwell invasion assay demonstrating the effect of conditioned media derived from GATA6/TET1-depleted CAFs and scramble siRNA-transfected CAFs (Control) on MDA-MB-231 breast cancer cell invasion. Scale bar: 100 µm. Quantitative analysis showed that MDA-MB-231 breast cancer cells incubated with conditioned media from CAFs transfected with siRNAs targeting GATA6 and TET1, or treated with SB 431542, exhibited a significantly lower invasion potential compared to control cells (*P < 0.05). Error bars represent standard deviation (SD). **D** Transwell invasion assay evaluating the effect of conditioned media derived from GATA6/TET1-overexpressing NFs and FLuc mRNA-treated NFs (Control) on MDA-MB-231 breast cancer cell invasion. Scale bar: 100 µm. Quantitative analysis revealed that MDA-MB-231 breast cancer cells incubated with conditioned media from CAFs transfected with GATA6 mRNA or TET1 mRNA exhibited a significantly higher invasion potential compared to control cells (*P < 0.05). Error bars represent standard deviation (SD). **E** Wound healing scratch assay showing the effect of conditioned media derived from GATA6/TET1-depleted CAFs and scramble siRNA-transfected CAFs (Control) on MDA-MB-231 breast cancer cell migration. Scale bar: 100 µm. Quantitative analysis showed that MDA-MB-231 breast cancer cells incubated with conditioned media from CAFs transfected with siRNAs targeting GATA6 and TET1, or treated with SB 431542, exhibited a significantly lower migration potential compared to control cells (*P < 0.05). Error bars represent standard deviation (SD). **F** Wound healing scratch assay illustrating the effect of conditioned media from GATA6/TET1-overexpressing NFs and FLuc mRNA-treated NFs (Control) on MDA-MB-231 breast cancer cell migration. Scale bar: 100 µm. Quantitative analysis revealed that MDA-MB-231 breast cancer cells incubated with conditioned media from CAFs transfected with GATA6 mRNA or TET1 mRNA exhibited a significantly higher migration potential compared to control cells (*P < 0.05). Error bars represent standard deviation (SD). Error bars represent standard deviation (SD).

To investigate the functional roles of GATA6 and TET1 in CAFs, we conducted loss-of-function (siRNA-mediated knockdown) and gain-of-function (mRNA overexpression) experiments. We demonstrated that transfecting normal breast fibroblasts (NFs) with GATA6 mRNA or TET1 mRNA significantly increased their respective protein levels compared to NFs transfected with firefly luciferase (FLuc) control mRNA (Control 1, *p* < 0.05; Fig. 2A, B). Conversely, siRNA-mediated knockdown of GATA6 (siRNA-GATA6) or TET1 (siRNA-TET1) in CAFs effectively reduced protein expression relative to scramble siRNA-transfected CAFs (Control 2, *p* < 0.001; Fig. 2A, B). To determine the functional consequence of GATA6/TET1 modulation, we measured the expression of other CAF-specific markers. Results demonstrated that downregulating GATA6 or TET1 via siRNA reduced protein levels of canonical CAF markers—α-smooth muscle actin (αSMA), fibroblast activation protein (FAP), and fibroblast-specific protein-1 (FSP-1)—compared to scrambled siRNA controls (*p* < 0.05; Fig. 2C, D). In contrast, overexpressing GATA6 or TET1 in NFs upregulated CAF marker expression and induced CAF-like phenotypes (*p* < 0.01 *vs*. FLuc-transfected NFs; Fig. 2C, E). We further assessed the impact of these treatments on CAF abundance. Knockdown of GATA6 and TET1 in CAFs decreased their population compared to scrambled siRNA-treated CAFs (Fig. 2F). Conversely, overexpressing GATA6 or TET1 in NFs via mRNA transfection increased the number of CAF-like cells (Fig. 2G). We further investigated the effects of GATA6 and TET1 on CAF cell survival, a critical aspect for therapeutic applications given the availability of chemical inhibitors targeting these genes. Our results demonstrated that knockdown of GATA6 and TET1 using specific siRNAs, or treatment with SB 431542 (SB), substantially increased CAF cell death compared to scrambled siRNA controls (*p* < 0.01; Fig. 3A). Moreover, we investigated the protein levels of cytokines and growth factors secreted by CAFs, including IL-6, VEGF, and TGF-β. Our data revealed that CAFs with reduced GATA6/TET1 exhibited decreased secretion of IL-6, VEGF, and TGF-β (*p* < 0.05 *vs*. Control 1; Fig. 3B), critical mediators of angiogenesis and tumor-stroma crosstalk. Conversely, overexpression of GATA6/TET1 in NFs enhanced secretion of these factors, compared to NFs transfected with the FLuc control template (*p* < 0.01 *vs*. Control 2; Fig. 3B). To further explore the impact of GATA6 and TET1 on CAF function, we conducted scratch and transwell assays to evaluate their effect on cancer cell migration and invasion. Results showed that conditioned media from GATA6/TET1-depleted CAFs reduced migration and invasion of MDA-MB-231 breast cancer cells (*p* < 0.001 *vs*. Control 1; Fig. 3C–F). Conversely, media from GATA6/TET1-overexpressing fibroblasts enhanced cancer cell aggressiveness, compared to fibroblasts transfected with the FLuc control template (Control 2) (*p* < 0.01; Fig. 3C–F). Overall, our findings demonstrate that GATA6 and TET1 are critical regulators of CAF identity, survival, and tumor-promoting functions. These results highlight GATA6 and TET1 as potential therapeutic targets to disrupt CAF-mediated tumor progression.

### TET1 regulates SMAD4 expression in breast cancer-associated fibroblasts via DNA demethylation

**Fig. 4:**
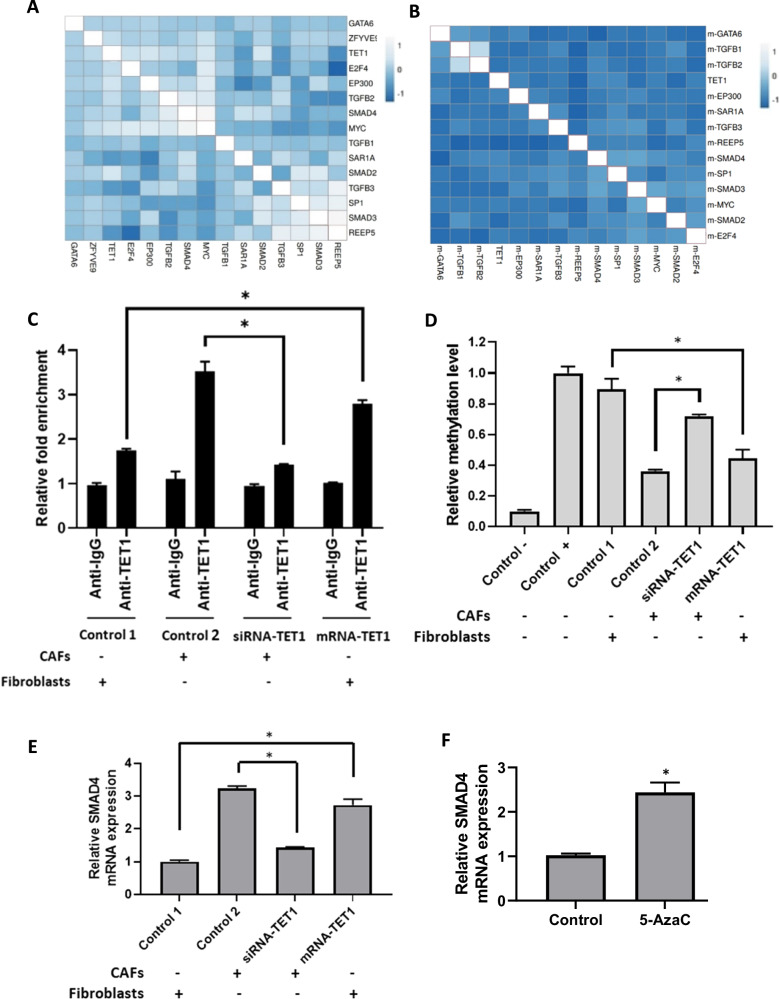
TET1 regulates SMAD4 expression via DNA demethylation in breast cancer-associated fibroblasts. **A** Correlation heatmap showing the positive association between TET1, GATA6, and TGF-β/SMAD pathway gene expression (positive correlation > 0; *P* < 0.05). Data were obtained from TCGA and METABRIC cohorts (n = 1986). **B** Correlation heatmap showing the negative association between TET1 expression and promoter methylation levels of TGF-β/SMAD pathway genes (negative correlation *<* 0; *P* < 0.05). Data were obtained from TCGA and METABRIC cohorts (*n* = 1986). **C** Chromatin immunoprecipitatio*n* (ChIP) assay demonstrating TET1 binding to the SMAD4 promoter in fibroblasts transfected with TET1 mRNA versus NFs transfected with FLuc mRNA (Control 1), and in CAFs transfected with TET1 siRNA versus scramble siRNA-treated CAFs (Control 2). IgG antibody (Anti-IgG) served as a negative control (*P < 0.05). **D** Methylation-sensitive high-resolution melting (MS-HRM) analysis of *SMAD4* promoter methylation in fibroblasts transfected with TET1 mRNA versus FLuc mRNA-treated NFs (Control 1), and in CAFs transfected with TET1 siRNA versus scramble siRNA-transfected CAFs (Control 2). Synthetic non-methylated (Control -) and methylated (Control+) templates were used as technical controls (*P < 0.05). **E** QRT-PCR analysis of SMAD4 mRNA expression in fibroblasts transfected with TET1 mRNA versus FLuc mRNA-treated NFs (Control 1), and in CAFs transfected with TET1 siRNA versus scramble siRNA-treated CAFs (Control 2) (*P < 0.05). **F** QRT-PCR analysis of SMAD4 mRNA expression in NFs treated with 5-azacytidine (5-AzaC) versus untreated NFs (*P < 0.05). Error bars represent standard deviation (SD).

While we proposed the functional importance of GATA6 and TET1 in CAF biology, the molecular mechanisms underlying their effects remained unclear. We hypothesized that TET1 drives CAF activation by inducing DNA demethylation at promoters of SMAD/TGF-β pathway genes, thereby enhancing their expression. To investigate the role of TET1 in regulating SMAD/TGF-β pathway genes in CAFs, we analyzed the correlation between promoter DNA methylation and gene expression of the SMAD/TGF-β pathway in relation to TET1 expression in breast cancer. In breast cancer cohorts (TCGA, METABRIC; *n* = 1,986), TET1 expression positively correlated with genes in the SMAD/TGF-β pathway, particularly SMAD4 (*P* < 0.05; Fig. 4A). Additionally, TET1 expression inversely correlated with DNA methylation levels at the SMAD4 promoter (*P* < 0.05; Fig. 4B). Accordingly, SMAD4 was selected as a key gene due to its multiple CpG islands in the promoter region and its critical role in the regulation of the SMAD/TGF-β pathway. Interestingly, chromatin immunoprecipitation (ChIP) revealed enriched TET1 binding at the SMAD4 promoter in CAFs compared to normal fibroblasts (NFs). Overexpression of TET1 mRNA in NFs increased promoter occupancy, while siRNA-mediated TET1 knockdown in CAFs reduced binding (*P* < 0.01 *vs*. controls; Fig. 4C). We also measured the DNA methylation status of the SMAD4 promoter using Methylation-Sensitive High-Resolution Melting (MS-HRM). Results demonstrated that DNA methylation levels of the SMAD4 promoter were higher in normal fibroblasts and CAFs transfected with TET1 siRNA, whereas DNA methylation levels were lower in CAFs and fibroblasts overexpressing TET1 mRNA, (P < 0.001 *vs*. controls; Fig. 4D). Additionally, we found that SMAD4 mRNA levels mirrored TET1 activity—knockdown reduced, and overexpression increased, SMAD4 expression (*P* < 0.05; Fig. 4E). These findings were further confirmed with the use of 5-azacytidine (5-AzaC), a DNA methylation inhibitor. Results showed that treatment with 5-AzaC phenocopied TET1 overexpression by upregulating SMAD4 expression (*P* < 0.01; Fig. 4F). Collectively, these results indicate that TET1 promotes SMAD4 expression in CAFs by demethylating its promoter, establishing a direct mechanistic link between TET1-mediated epigenetic remodeling and TGF-β/SMAD pathway activation. This axis is critical for maintaining CAF identity and function.

### SMAD4-mediated GATA6 expression regulates TGF-β expression in breast cancer-associated fibroblasts

**Fig. 5:**
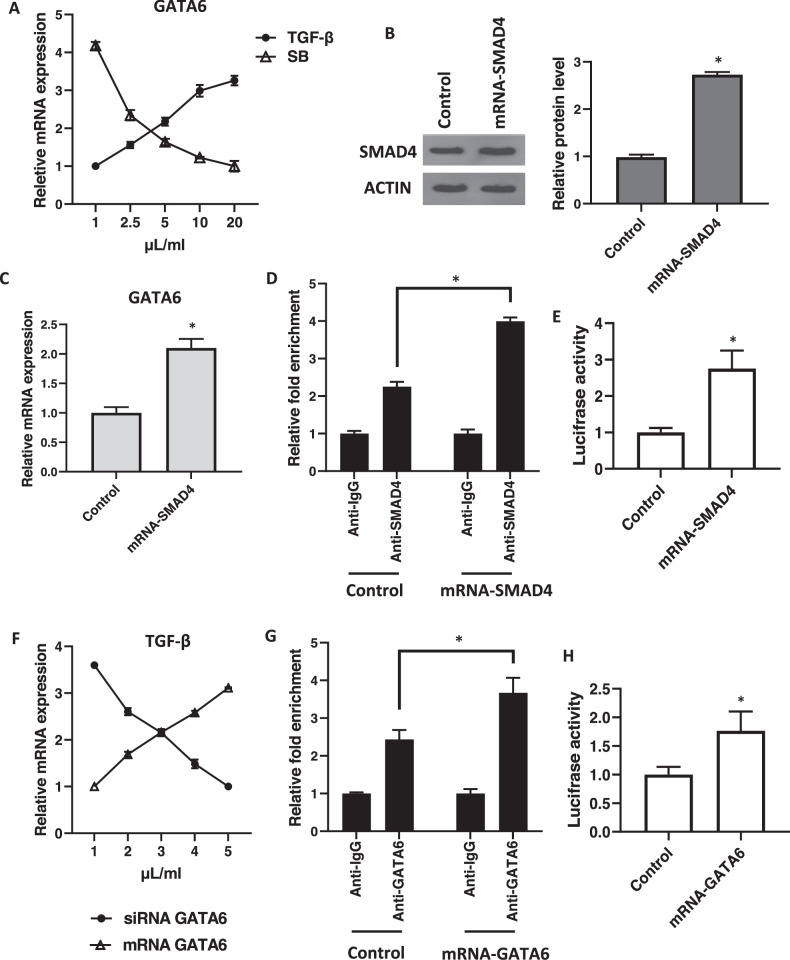
SMAD4-mediated GATA6 regulates TGF-β in breast cancer-associated fibroblasts. **A** QRT-PCR analysis of GATA6 expression in NFs treated with increasing concentrations of TGF-β versus untreated NFs, and in CAFs treated with increasing concentrations of SB 431542 (SB) versus untreated CAFs. **B** Western blot analysis of SMAD4 protein levels in NFs transfected with SMAD4 mRNA versus FLuc mRNA-treated NFs (Control). ACTIN served as a loading control. The cropped blots are presented correspondingly. Full-length blots are presented in Supplementary Fig. 4. For the quantitative analysis of SMAD4 protein level, band density was assessed using ImageJ software and represented as relative intensity. *P < 0.05. **C** QRT-PCR analysis of GATA6 expression in NFs transfected with SMAD4 mRNA versus FLuc mRNA-treated NFs (Control) (*P < 0.05). **D** Chromatin immunoprecipitation (ChIP) assay demonstrating SMAD4 binding to the GATA6 promoter in fibroblasts transfected with SMAD4 mRNA versus FLuc mRNA-treated NFs (Control). IgG antibody (Anti-IgG) served as a negative control (*P < 0.05). **E** Luciferase assay showing the effect of SMAD4 on GATA6 promoter activity (*P < 0.05). **F** QRT-PCR analysis showing the correlation between TGF-β and GATA6 expression. TGF-β expression increased in NFs transfected with increasing concentrations of GATA6 mRNA compared to FLuc mRNA-treated NFs, and decreased in CAFs transfected with increasing concentrations of GATA6 siRNA compared to scramble siRNA-transfected CAFs (*P < 0.05). **G** ChIP assay demonstrating GATA6 binding to the TGF-β promoter in fibroblasts transfected with GATA6 mRNA compared to FLuc mRNA-treated NFs (Control). IgG antibody (Anti-IgG) served as a negative control (*P < 0.05). **H** Luciferase assay showing the direct regulation of TGF-β promoter activity by GATA6 (*P < 0.05). Error bars represent standard deviation (SD).

Building on our findings regarding TET1’s role in CAFs, we hypothesized that GATA6 regulates TGF-β expression, as siRNA-mediated knockdown of GATA6 significantly reduced TGF-β protein levels in CAFs. To explore this, we analyzed the correlation between GATA6 expression and the TGF-β pathway genes in breast cancer cohorts (TCGA, METABRIC; *N* = 1,986). Data revealed that GATA6 expression positively correlated with TGF-β and SMAD4 expression (*P* < 0.05; Fig. 4A), suggesting a regulatory link. We then evaluated the mechanism through which TGF-β/SMAD4 Drives GATA6 Expression. Our observations indicated that treatment of normal fibroblasts (NFs) with TGF-β progressively increased GATA6 mRNA expression, while inhibition of TGF-β signaling with SB 431542 (SB) in CAFs reduced GATA6 transcript levels (*P* < 0.01; Fig. 5A). Consistently, overexpression of SMAD4 in fibroblasts significantly upregulated GATA6 expression (*P* < 0.001 *vs*. FLuc control; Fig. 5B, C). Importantly, ChIP confirmed enhanced binding of SMAD4 to the GATA6 promoter upon SMAD4 overexpression (*P* < 0.01; Fig. 5D). Luciferase assays showed that overexpression of SMAD4 significantly enhanced GATA6 promoter activity, further confirming the direct effect of SMAD4 on GATA6 expression (P < 0.05; Fig. 5E). In order to assess whether GATA directly mediates TGF-β expression, we assessed the effect of GATA6 expression on TGF-β levels. Our data showed that overexpression of GATA6 in NFs increased TGF-β mRNA levels, while GATA6 knockdown in CAFs reduced TGF-β expression (*P* < 0.01; Fig. 5F). ChIP assays revealed stronger binding of GATA6 to the TGF-β promoter in CAFs compared to NFs (*P* < 0.001; Fig. 5G). We then conducted a luciferase assay to assess whether GATA6 directly interacts with TGF-β. Results indicated that GATA6 overexpression significantly enhanced TGF-β promoter activity (*P* < 0.01; Fig. 5H). These findings establish a self-reinforcing regulatory loop where SMAD4 and GATA6 mutually reinforce each other’s activity, highlighting their critical roles in the CAF-mediated tumor microenvironment.

### GATA6 and TET1 knockdown reduces breast cancer progression in vivo

**Fig. 6:**
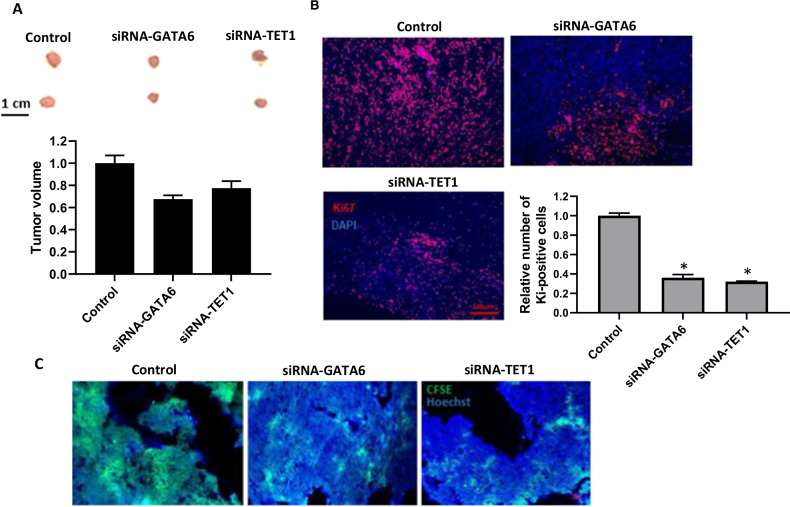
Knockdown of GATA6 and TET1 suppresses CAF-mediated tumor progression. **A**. Tumor volume in MDA-MB-231 breast cancer cell xenografts co-injected with CAFs transfected with siRNA-GATA6 or siRNA-TET1 versus scramble siRNA-treated CAFs (Control). Scale bar: 1 cm. **B** Immunofluorescence analysis of Ki67^+^ cells in tumors derived from CAFs transfected with siRNA-GATA6 or siRNA-TET1 versus scramble siRNA-treated CAFs (Control). Scale bar: 100 µm. Quantitative analysis shows the *relative number of Ki67+ cells* in each group (*P < 0.05). **C** CFSE signal intensity in tumors derived from CFSE-labeled CAFs transfected with siRNA-GATA6 or siRNA-TET1 versus scramble siRNA-treated CAFs (Control). Scale bar: 100 µm. Error bars represent standard deviation (SD). All statistical tests had a post hoc power >80%.

To evaluate the role of GATA6 and TET1 in CAF-mediated tumor progression in vivo, we established a xenograft model using CFSE-labeled CAFs co-injected with breast cancer cells into nude mice. CAFs were transfected with siRNA targeting GATA6 or TET1 prior to injection, while scramble siRNA-transfected CAFs served as controls. After seven days of injection, when tumors were visible, the mice underwent further experimentation. We found that tumor size in mice injected with GATA6- or TET1-depleted CAFs were ~20% smaller than those in the control group (Mice transplanted with scramble siRNA-transfected CAFs) (*P* < 0.05; Fig. 6A). Immunohistochemical staining revealed a significant reduction in Ki67-positive cells in tumors derived from GATA6- or TET1-depleted CAFs (*P* < 0.01 *vs*. control; Fig. 6B), indicating suppressed tumor cell proliferation. Also, CFSE signal intensity, reflecting CAF persistence, was markedly lower in tumors from GATA6- or TET1-depleted CAFs (*P* < 0.001 *vs*. control; Fig. 6C), suggesting reduced CAF survival and engraftment. These results demonstrate that GATA6 and TET1 are critical for CAF-mediated tumor progression in vivo, influencing both tumor growth and stromal-tumor interactions.

## Discussion

The TME is a critical orchestrator of solid tumor initiation, progression, and metastasis. Interactions between tumor cells and stromal components—such as fibroblasts, immune cells, extracellular matrix (ECM) proteins, and vasculature—profoundly influence tumor behavior [32]. Fibroblasts, typically quiescent guardians of tissue homeostasis, undergo dynamic activation in response to TME-derived signals, transdifferentiating into CAFs. This transformation disrupts normal fibroblast signaling pathways [33], culminating in a heterogenous CAF population characterized by the secretion of ECM proteins, growth factors, cytokines, and remodeling enzymes that collectively fuel tumor growth, invasion, and metastasis. CAFs may originate from diverse cellular sources, including resident fibroblasts, epithelial cells, endothelial cells, and bone marrow-derived mesenchymal stem cells, contributing to their functional plasticity and complicating their role in cancer biology [30]. The conversion of normal fibroblasts (NFs) to CAFs is a multi-step process regulated by key pathways such as TGF-β. CAFs and tumor cells engage in reciprocal crosstalk, forming a self-reinforcing feedback loop that drives tumor progression while shaping CAF differentiation and function. Beyond tumor promotion, CAFs remodel the immune landscape, enhance angiogenesis, and confer therapy resistance, positioning them as pivotal therapeutic targets [34]. Unraveling the signaling pathways and transcription factors governing CAF induction and maintenance is essential for developing strategies to neutralize their pro-tumorigenic activities. Targeting these molecular regulators could yield therapies that reprogram CAFs or disrupt their crosstalk with tumor cells, offering promise for improving treatment efficacy and mitigating metastasis and relapse [30, 35].

**Fig. 7:**
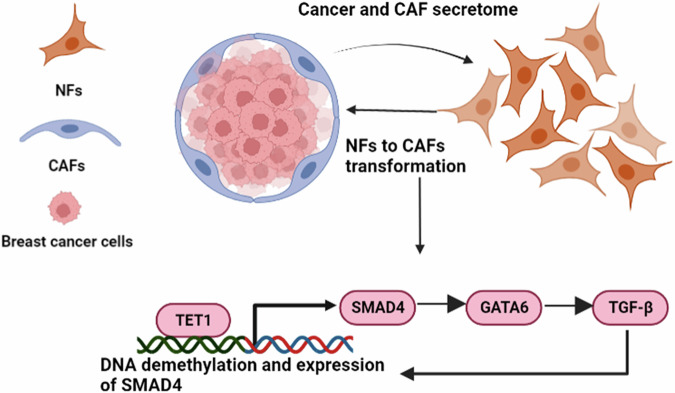
Proposed mechanism of GATA6 and TET1 in regulating CAF transformation and breast cancer progression. In this study, we identified the TET1/SMAD4/GATA6 regulatory axis as a central driver of CAF activation. Mechanistically, TET1 demethylates the SMAD4 promoter, enhancing SMAD4 expression. SMAD4, in turn, transcriptionally upregulates GATA6, which amplifies TGF-β signaling by directly activating the TGF-β promoter. This feedforward circuit sustains CAF identity, fosters stromal-tumor crosstalk, and perpetuates a pro-tumorigenic microenvironment. Overall, our findings demonstrate that GATA6 and TET1 are critical regulators of CAF identity, survival, and tumor-promoting functions, highlighting their potential as therapeutic targets to disrupt CAF-mediated tumor progression.

In this study, we identified the TET1/SMAD4/GATA6 regulatory axis as a central driver of CAF activation. Our analysis of expression data from NFs and CAFs implicated epithelial differentiation pathways in CAF transformation. Mechanistically, TET1 demethylates the *SMAD4* promoter, enhancing SMAD4 expression. SMAD4, in turn, transcriptionally upregulates *GATA6*, which amplifies TGF-β signaling by directly activating the *TGF-β* promoter. This feedforward circuit sustains CAF identity, fosters stromal-tumor crosstalk, and perpetuates a pro-tumorigenic microenvironment (Fig. 7). GATA6, a transcription factor critical for epithelial differentiation during embryogenesis, emerged as a key CAF regulator. Its elevated expression in CAFs aligns with its roles in myofibroblast activation, fibrosis, and tumor progression [36, 37]. While GATA6’s involvement in pancreatic cancer and EMT regulation via Slug in breast cancer is established [20, 38], its functions in CAFs remain underexplored. Similarly, TET1, an epigenetic modulator overexpressed in CAFs, reshapes the CAF epigenome by hypomethylating oncogenic pathways (e.g., PI3K, EGFR, PDGF) in triple-negative breast cancer [39]. Despite their known roles in cellular transformation, the CAF-specific mechanisms of GATA6 and TET1 require deeper investigation.

Our functional studies demonstrated that GATA6 and TET1 govern CAF identity, proliferation, and pro-tumorigenic activity. GATA6 overexpression elevated TGF-β secretion, CAF marker expression (e.g., αSMA), and CAF abundance, while its knockdown diminished these effects. GATA6’s interaction with SMAD4, which binds the *TGF-β* promoter, suggests a novel autocrine loop linking SMAD4 and TGF-β—a mechanism previously observed in fibrosis and stem cell niches [40, 41]. Similarly, TET1 depletion reduced CAF survival, marker expression, and ability to drive cancer cell migration/invasion. TET1’s demethylation of the *SMAD4* promoter implicates epigenetic remodeling in TGF-β pathway activation, though further validation is needed. Knockdown of GATA6 or TET1 attenuated CAF-mediated tumor growth in vivo, underscoring their potential as therapeutic targets. Tumors derived from GATA6- or TET1-depleted CAFs exhibited reduced size, proliferation (Ki67^+^ cells), and CAF engraftment (CFSE signal). These findings align with the efficacy of TGF-β inhibition (e.g., SB 431542) in suppressing CAF activity, highlighting the centrality of this pathway. While CAF inhibition holds therapeutic promise, challenges such as stromal heterogeneity and CFSE signal dilution over successive cell divisions warrant deeper mechanistic exploration. CFSE enables high-resolution, short-term tracing of CAF proliferation dynamics in vivo; however, its utility is constrained by fluorescence decay. This limitation necessitated a 7-day endpoint to prioritize capturing early tumor-stroma interactions, where stromal influence is most pronounced. Although this precluded long-term tumor size quantification, CFSE’s sensitivity aligns with established murine cell-tracking protocols [42, 43], allowing precise measurement of clonal expansion and spatial redistribution—capabilities often lost with slower-decaying labels. Future studies integrating longitudinal bioluminescence imaging could bridge early CFSE-derived insights with later tumor progression. Together, these findings highlight CFSE’s unique role in dissecting acute cellular behaviors, providing a framework for temporally precise investigations of microenvironmental crosstalk

## Conclusion

In summary, our study elucidates the critical roles of GATA6 and TET1 in sustaining CAF function and driving breast cancer progression. These regulators operate within a TGF-β/SMAD4-dependent axis, where TET1-mediated epigenetic remodeling and GATA6-driven transcriptional activation amplify TGF-β signaling. Targeting GATA6 or TET1 disrupted CAF identity, impaired tumor growth, and attenuated stromal-tumor crosstalk in vitro and in vivo. These findings position GATA6 and TET1 as promising therapeutic targets, particularly in CAF-rich malignancies like breast cancer. Future studies should refine strategies to exploit this axis, addressing mechanistic nuances and optimizing translational potential.

## Materials and methods

### Clinical samples

Tumoral and adjacent non-tumoral tissues (*n* = 10 per group) were obtained from fresh surgical specimens of breast cancer patients. Clinicopathological characteristics of the patients are summarized in Supplementary Table 1. Tissues were collected in transport media (PBS supplemented with 1% penicillin-streptomycin; Gibco) and immediately transported on ice to the cell culture laboratory.

### Isolation of stromal fibroblasts and cell culture

Primary breast tumor tissues were obtained from three female patients with histological grade III invasive ductal carcinoma who underwent mastectomy. For processing, tissues were washed twice with PBS containing 2% penicillin-streptomycin and 1% amphotericin B (Gibco), minced into 1–2 mm^3^ fragments, and enzymatically digested in a solution of collagenase type I (1 mg/mL) and 0.25% trypsin-EDTA (Gibco) for 30 min at 37 °C. The digestion mixture was gently shaken every 10 min to ensure uniform tissue dissociation. The cell suspension was centrifuged at 300 × *g* for 5 min, and the pellet was resuspended in high-glucose DMEM (Gibco) supplemented with 20% fetal bovine serum (FBS; Gibco) and 1% penicillin-streptomycin. Isolated cells were cultured in a humidified 5% CO_2_ incubator at 37 °C. After 24 h, the medium was replaced with high-glucose DMEM containing 10% FBS and 1% penicillin-streptomycin. At 80–90% confluency, cells were passaged using 0.25% trypsin-EDTA and reseeded for subculture [44]. To obtain induced CAFs, normal fibroblasts were treated with 10 ng/ml TGF-β (Sigma, USA) for 72 h. For TGF-β/SMAD pathway inhibition, cells were pre-treated with 0.3 μM SB 431542 (SB; Sigma, USA) for 72 h prior to analysis. MDA-MB-231 cells were cultivated in high-glucose DMEM supplemented with 10% FBS and 1% penicillin-streptomycin. For epigenetic modulation, fibroblasts were treated with 1.5 μg/ml 5-azacytidine (5-AzaC; Sigma, USA) for 72 h.

### In vitro transient transfection

For each transfection, 2.5 μL of siRNA or mRNAs (10 μM stock; sequences listed in Supplementary Table 2) was diluted in 50 μL Opti-MEM Reduced Serum Medium (Thermo Fisher, USA). Separately, 25 μL Lipofectamine RNAiMAX transfection reagent (Thermo Fisher, USA) was mixed with 50 μL Opti-MEM. The diluted RNA and transfection reagent solutions were combined, gently mixed, and incubated at room temperature for 15 min to allow transfection complex formation. The RNA-lipid complexes (total volume: 50 μL) were added dropwise to cells seeded in a 24-well plate (70% confluency) containing 1 mL complete medium (DMEM with 10% FBS and 1% penicillin-streptomycin), following the manufacturer’s protocol. Cells were incubated with the siRNA for three days before performing the assay. Cells were maintained in the transfection mixture for 72 h at 37 °C in a 5% CO_2_ incubator prior to downstream analysis.

### Immunohistochemistry and immunocytochemistry

Collected tissue samples were fixed in 10% neutral buffered formalin for 24 h, dehydrated through a graded ethanol series, and embedded in paraffin. Paraffin-embedded blocks were sectioned into 4-μm-thick slices, mounted on glass slides, and rehydrated through xylene and a descending ethanol gradient. Sections were permeabilized with 0.1% Triton X-100 (Sigma, USA) and subjected to heat-mediated antigen retrieval in sodium citrate buffer (10 mM, pH 6.0) at 95 °C for 20 min. Slides or cultured cells (fixed in 4% ice-cold paraformaldehyde for 15 min) were blocked with 5% bovine serum albumin (BSA) in PBS for 1 h at room temperature. Samples were incubated overnight at 4 °C with primary antibodies (Supplementary Table 3) in blocking buffer. After three washes with PBS, species-matched fluorescent secondary antibodies (1:500; Supplementary Table 3) were applied for 1 h at room temperature. Nuclei were counterstained with 300 nM DAPI (Sigma, USA) for 5 min. Stained samples were imaged using an Olympus BX51 fluorescence microscope equipped with appropriate excitation/emission filters. For quantification, three biological replicates (with three technical replicates each) were analyzed. Positively stained cells were quantified using ImageJ software by calculating fluorescence intensity or percentage of DAPI-positive cells relative to controls.

### Western blot

Cellular proteins were extracted using RIPA lysis buffer (Thermo Fisher, USA). Protein lysates were resolved by SDS-PAGE on a 10% gel and electrophoresed at a constant voltage (100 V). Separated proteins were transferred to a PVDF membrane (Millipore, USA) using a semi-dry transfer system. The membrane was blocked with 5% non-fat skim milk in TBST (Tris-buffered saline with 0.1% Tween-20) for 1 h at room temperature. Subsequently, the membrane was incubated overnight at 4 °C with primary antibodies (Supplementary Table 3). After three 5-min washes with TBST, species-matched horseradish peroxidase (HRP)-conjugated secondary antibodies (1:10,000; Supplementary Table 3) were applied and incubated for 1 h at room temperature. Following three additional TBST washes, chemiluminescent signals were detected using a Chemiluminescent HRP Substrate (Millipore, USA; Solutions A and B mixed at a 1:1 ratio) and imaged with a ChemiDoc system (Bio-Rad, USA). Protein band intensities were quantified by densitometry using ImageJ software and normalized to β-actin as a loading control.

### Migration and invasion assays

To perform transwell invasion assay, Matrigel (Corning, USA) was diluted 1:3 in serum-free DMEM, and 120 μL of the mixture was evenly coated onto the upper chamber of a 24-well transwell insert (8-μm pore size; Corning, USA). After polymerization at 37 °C for 6 h, 1 × 10^5^ MDA-MB-231 cells in serum-free medium were seeded into the upper chamber. Conditioned medium harvested from fibroblast cultures was added to the lower chamber as a chemoattractant. Following a 24-h incubation, non-invading cells on the upper membrane surface were removed with a cotton swab. Invaded cells on the lower surface were fixed in methanol, stained with hematoxylin, and quantified by counting five random fields per insert under a light microscope (Olympus CX33). Experiments were performed in triplicate biological replicates. To perform wound healing scratch assay, MDA-MB-231 cells were seeded in 6-well plates and grown to 70–80% confluency. A uniform scratch wound was created using a 200-μL pipette tip. Debris was removed by washing with PBS, and fibroblast-conditioned medium was added to the wells. Wound closure was monitored at 6-h intervals using phase-contrast microscopy (Olympus CX33). Migration was quantified by measuring the remaining scratch area at each time point using ImageJ software [45].

### RNA extraction and qRT-PCR

Total RNA was extracted from tissues and cells with TRI reagent (Sigma, USA). Briefly, 1 × 10^6^ cells or 1 mg of homogenized tissues were lysed in 1 mL of TRI reagent and incubated for 15 min at room temperature. Chloroform (200 μL) was added, and the mixture was vortexed vigorously for 15 s, followed by a 10-min incubation. After centrifugation at 12,000 × *g* for 15 min at 4 °C, the aqueous phase was transferred to a new tube, mixed with 0.5 mL isopropanol, and centrifuged at 12,000 × *g* for 10 min to pellet RNA. The RNA pellet was washed twice with 75% ethanol, air-dried, and resuspended in 20 μL RNase-free TE buffer (10 mM Tris-HCl, 1 mM EDTA, pH 8.0). RNA purity and concentration were assessed using a NanoDrop 2000 spectrophotometer (Thermo Fisher, USA), and integrity was confirmed by 1% agarose gel electrophoresis. For cDNA synthesis, 1 μg of total RNA was reverse-transcribed using PrimeScript™ IV Reverse Transcriptase (Takara, Japan) in a 20 μL reaction containing 4 μL 5× PrimeScript IV Buffer, 1 μL Oligo(dT) primer, 1 μL random hexamers, and nuclease-free water. Reactions were incubated at 30 °C for 10 min (primer annealing), 42 °C for 20 min (cDNA synthesis), and 95 °C for 5 min (enzyme inactivation). QRT-PCR was performed using SYBR® Premix Ex Taq™ (Takara, Japan) on an ABI Step One Detection System (Applied Biosystems, USA). Primer used for qRT-PCR are listed in Supplementary Table 2. Thermal cycling conditions were: 95 °C for 30 s, followed by 40 cycles of 95 °C for 5 s and 60 °C for 30 s. Relative mRNA expression was calculated using the ΔΔCt method [46], normalized to β-actin as a housekeeping gene, and reported as fold change relative to controls.

### Hoechst 33342 and propidium iodide double staining

Cells were seeded in 24-well plates and allowed to reach 50–60% confluency. The culture medium was aspirated, and cells were incubated with 0.1 µg/mL Propidium Iodide (PI; Sigma, USA) in PBS for 10 min at 37 °C to label dead/damaged cells. After two washes with PBS, cells were stained with 0.1 µg/mL Hoechst 33342 (Sigma, USA) for 5 min at 37 °C to visualize all nuclei. After two additional PBS washes, cells were resuspended in PBS and immediately imaged using an Olympus BX51 fluorescence microscope equipped with Hoechst and propidium iodide (PI)**-**specific excitation/emission filters. For quantification, the total number of Hoechst-positive nuclei and PI-positive cells (indicative of membrane compromise) were counted in five random fields per well using ImageJ software.

### Chromatin immunoprecipitation and methylation-sensitive high-resolution melting

Chromatin immunoprecipitation (ChIP) assays were performed using the SimpleChIP® Enzymatic Chromatin IP Kit (Cell Signaling Technology, USA) according to the manufacturer’s protocol. Chromatin was crosslinked, enzymatically digested, and immunoprecipitated with antibodies targeting TET1, SMAD4, and GATA6 **(**Supplementary Table 3**)**. Normal rabbit IgG (Cell Signaling) served as a negative control. Precipitated DNA was purified and analyzed by qRT-PCR using primers specific to promoter regions of interest **(**Supplementary Table 2**)**, as described in the RNA extraction and qRT-PCR section. For Methylation-Sensitive High-Resolution Melting (MS-HRM), genomic DNA was isolated using TRI Reagent (Sigma, USA) and sodium bisulfite-modified using the EZ DNA Methylation-Gold Kit (Zymo Research, USA) according to the manufacturer’s protocol. Briefly, 1 μg of DNA was denatured with 1 μL of 6.3 M NaOH, incubated in a bisulfite solution (3.5 M sodium bisulfite, 0.5 mM hydroquinone, pH 5.1) for 16 h at 55 °C. Bisulfite-treated DNA was desulfonated with 0.3 M NaOH (pH 7.0), purified using the Wizard® DNA Clean-Up System (Promega, USA), and eluted in 20 μL Tris-EDTA buffer (10 mM Tris-HCl, 1 mM EDTA, pH 8.0). To perform MS-HRM analysis, qPCR was performed on a Rotor-Gene 5plex HRM System (Qiagen, Germany) in 20 μL reactions containing 10 μL SYBR Green Master Mix (PCRBIO, USA), 10 pmol primers **(**Supplementary Table 2**)**, and 50 ng bisulfite-converted DNA. Fully methylated and unmethylated synthetic DNA standards (PCRBIO, USA) were included as controls. Melting curve data were analyzed using Rotor-Gene Q Software (Qiagen) to quantify methylation levels [47].

### Luciferase assay

The pGL4-GATA6 and pGL4-TGF-β1 firefly luciferase reporter vectors were constructed by cloning the human GATA6 promoter and TGF-β1 promoter into the pGL4.10[luc2] backbone (Promega, USA), respectively. Normal fibroblasts were co-transfected in 24-well plates with 1 μg of pGL4-GATA6 or pGL4-TGF-β1, 500 ng SMAD4 mRNA, and 500 ng GATA6 mRNA using Lipofectamine 3000 (Thermo Fisher, USA). The empty pGL4.10[luc2] vector served as a negative control. After 48 h, luciferase activity was quantified using the Dual-Luciferase Reporter Assay System (Promega, USA) according to the manufacturer’s protocol. Firefly luciferase signals were normalized to Renilla luciferase (co-transfected pRL-TK vector, Promega) to account for transfection efficiency, and results were expressed as fold change relative to control.

### In vivo tumor xenograft experiments

Female immunodeficient nude mice were used for tumor xenograft studies [48]. For tumor induction, MDA-MB-231 cells (80% confluent in a 24-well plate) were firstly trypsinized, washed with PBS, and resuspended in cold PBS. Cells were mixed with CellTrace™ CFSE (carboxyfluorescein succinimidyl ester)-labeled CAFs at a 1:1 ratio (50,000 MDA-MB-231 cells + 50,000 siRNA- or scramble RNA- transfected CAFs) in 100 μL PBS. The cell suspension was injected into the fourth mammary fat pad of anesthetized mice (*n* = 2 per group). Palpable tumors were first observed at 7 days post-injection. Mice were euthanized at the study endpoint, and tumors were excised for analysis. This approach aligns with established methods for monitoring cell proliferation in murine models using CFSE, as previously described [42, 43, 49]. Tumor volume was calculated using the formula: Volume = (Length × Width²)/2. Excised tumors were fixed in 10% neutral buffered formalin, paraffin-embedded, and sectioned for histological and immunohistochemical analysis.

### In silico analysis

mRNA expression and methylation profiles of the TGF-β signaling pathway (KEGG pathway ID: hsa04350) were analyzed in breast cancer samples from the METABRIC cohort. RNA-seq data and methylation array data were integrated to assess Pearson correlation coefficients between pathway-associated genes. Heatmaps visualizing gene expression clusters were generated using ClustVis. To identify differentially expressed genes (DEGs) between normal fibroblasts (NFs) and cancer-associated fibroblasts (CAFs) in breast tissue, microarray dataset E-GEOD-29270 was retrieved from the ArrayExpress database [45]. Raw data were normalized using Robust Multi-array Average (RMA), and DEGs were analyzed with the iDEP.96 platform (v1.0). Statistical significance was defined as |log2 fold change|> 1.5 and adjusted *p* value < 0.05. Functional enrichment analysis of DEGs was performed using Gene Ontology (GO) and KEGG pathway databases.

### Statistical analysis

Statistical analyses were performed using GraphPad Prism 8.0 (GraphPad Software, USA). Data are presented as the mean ± standard deviation (SD). Student’s *t* test was used for two-group comparisons, and one-way or two-way ANOVA followed by Tukey’s post hoc test was used for multiple comparisons. All experiments included at least three independent biological replicates, and statistical significance was defined as *p* < 0.05. Post hoc power analysis (G*Power 3.1; α = 0.05, power = 0.8) confirmed that sample sizes were sufficient to detect effect sizes observed in preliminary studies.

## Supplementary information

Supplementary tables

Supplementary data- Full blots

## Data Availability

The data supporting the findings of this study are available from the corresponding author upon reasonable request.
