# Supplementary material for: The transcriptional regulators GATA6 and TET1 regulate the TGF-β pathway in cancer-associated fibroblasts to promote breast cancer progression
Source: Cell Death Discov. 2025 Apr 11;11:164. doi: 10.1038/s41420-025-02438-4 (PMC11992015; doi:10.1038/s41420-025-02438-4)
Supplement: Supplementary file 1 — Supplementary tables [file 41420_2025_2438_MOESM1_ESM.docx]

**Supplementary Tables**

**Supplementary Table 1. Clinical characteristics of the study cohort**

| Patient No.* | Age (Year) | Tumor stage | Lymph node metastasis | ER/PR | HER2 | Molecular subtype |
| --- | --- | --- | --- | --- | --- | --- |
| Sample 1 | >70 | III+IV | Yes | -/- | - | Triple negative |
| Sample 2 | >70 | III+IV | Yes | -/- | - | Triple negative |
| Sample 3 | <70 | III+IV | Yes | -/- | - | Triple negative |
| Sample 4 | >70 | III+IV | Yes | -/- | - | Triple negative |
| Sample 5 | <70 | III+IV | Yes | -/- | + | HER2 Enriched |
| Sample 6 | <70 | II | No | -/- | + | HER2 Enriched |
| Sample 7 | >70 | III+IV | Yes | +/+ | - | Luminal A |
| Sample 8 | <70 | II | No | +/+ | - | Luminal A |
| Sample 9 | >70 | III+IV | Yes | +/+ | - | Luminal A |
| Sample 10 | <70 | II | Yes | +/+ | + | Luminal B |

*Non-tumoral tissues were paired with tumoral samples from the same patients

**Supplementary Table 2. Oligonucleotide sequences used in this study**

|  | **Sequence name** | **Primer sequence** |
| --- | --- | --- |
| **QRT-PCR** | |  |
|  | TET1-primer | Forward: 5’-CATCAGTCAAGACTTTAAGCCCT-3’ |
|  | | Reverse: 5’-CGGGTGGTTTAGGTTCTGTTT-3’ |
|  | GATA6-primer | Forward: 5’-GAGGTTCCCGAAACCA-3’ |
|  | | Reverse: 5’-AAAAGGAGAGAGGAGGCG-3’ |
|  | TGF-β primer | Forward: 5’-GACACCAACTATTGCTTCAG-3’ |
|  | | Reverse: 5’-CAGGCTCCAAATGTAGGG-3’ |
|  | SMAD4 primer | Forward: 5’-CCAATCATCCTGCTCCTGAGT-3’ |
|  | | Reverse: 5’-CCAGAAGGGTCCACGTATCC-3’ |
| **siRNA sequences** | |  |
|  | siRNA-TET1 | CCGGACACAACTTGCTTCGAT |
|  | siRNA-GATA6 | CTCCCCGGTCTACGTGCCCAC |
|  | scramble siRNA | ATCGAAGCAAGTTGTGTCCGG |
| **mRNA oligonucleotide** | | **Accession No.** |
|  | TET1 | NM_001406365.1 |
|  | GATA6 | NM_005257.6 |
|  | SMAD4 | NM_005359.6 |
| **ChIP-qPCR** | |  |
|  | TGF-β | Forward: 5’-TGGGATACTGAGACACCCCC-3’ |
|  | | Reverse: 5’-TTCACCAGCTCCATGTCGAT-3’ |
|  | GATA6 | Forward: 5’-CTCCGACGGTGTTTCCC-3’ |
|  | | Reverse: 5’-AAGTTTCTCTGCCTGCCTAACTA-3’ |
|  | SMAD4 | Forward: 5’-AACGAGATGCCAATTTCC-3’ |
|  | | Reverse: 5’-GTGTTGTTGCTGCCAACTT-3’ |
| **MS-HRM** | |  |
|  | SMAD4 | Forward: 5’-AACGAGATGCCAATTTCC-3’ |
|  | | Reverse: 5’-GTGTTGTTGCTGCCAACTT-3’ |

**Supplementary Table 3. List of antibodies used in this study**

| **Antibody** | **Company** | **Cat No.** |
| --- | --- | --- |
| Smooth Muscle Actin (CGA7) | Santa Cruz Biotechnology | sc-53015 |
| β-Actin (C4) | Santa Cruz Biotechnology | sc-47778 |
| FAP | Elabscience | E-AB-32870 |
| GATA-6 (F-3) | Santa Cruz Biotechnology | sc-518050 |
| IL-6 (E-4) | Santa Cruz Biotechnology | sc-28343 |
| AIFM2/ FSP1 | Thermo Fisher Scientific | 20886-1-AP |
| TET1 (4F4) | Santa Cruz Biotechnology | sc-293186 |
| Vimentin (V9) | Thermo Fisher Scientific | 14-9897-82 |
| VEGF (C-1) | Santa Cruz Biotechnology | sc-7269 |
| TGFβ1 (V) | Santa Cruz Biotechnology | sc-146 |
| PDGFR-β (18A2) | Santa Cruz Biotechnology | sc-19995 |
| Goat anti-mouse IgG-PE | Santa Cruz Biotechnology | sc-3738 |
| Goat Anti-Rabbit IgG(H+L) (FITC) | Biorbyt | orb688925 |
| m-IgGκ BP-HRP | Santa Cruz Biotechnology | sc-516102 |
| mouse anti-rabbit IgG-HRP | Santa Cruz Biotechnology | sc-2357 |
| Ki-67 | Santa Cruz Biotechnology | sc-23900 |
