# Supplementary material for: The transcriptional regulators GATA6 and TET1 regulate the TGF-β pathway in cancer-associated fibroblasts to promote breast cancer progression
Source: Cell Death Discov. 2025 Apr 11;11:164. doi: 10.1038/s41420-025-02438-4 (PMC11992015; doi:10.1038/s41420-025-02438-4)
Supplement: Supplementary file 2 — Supplementary data- Full blots [file 41420_2025_2438_MOESM2_ESM.pdf]

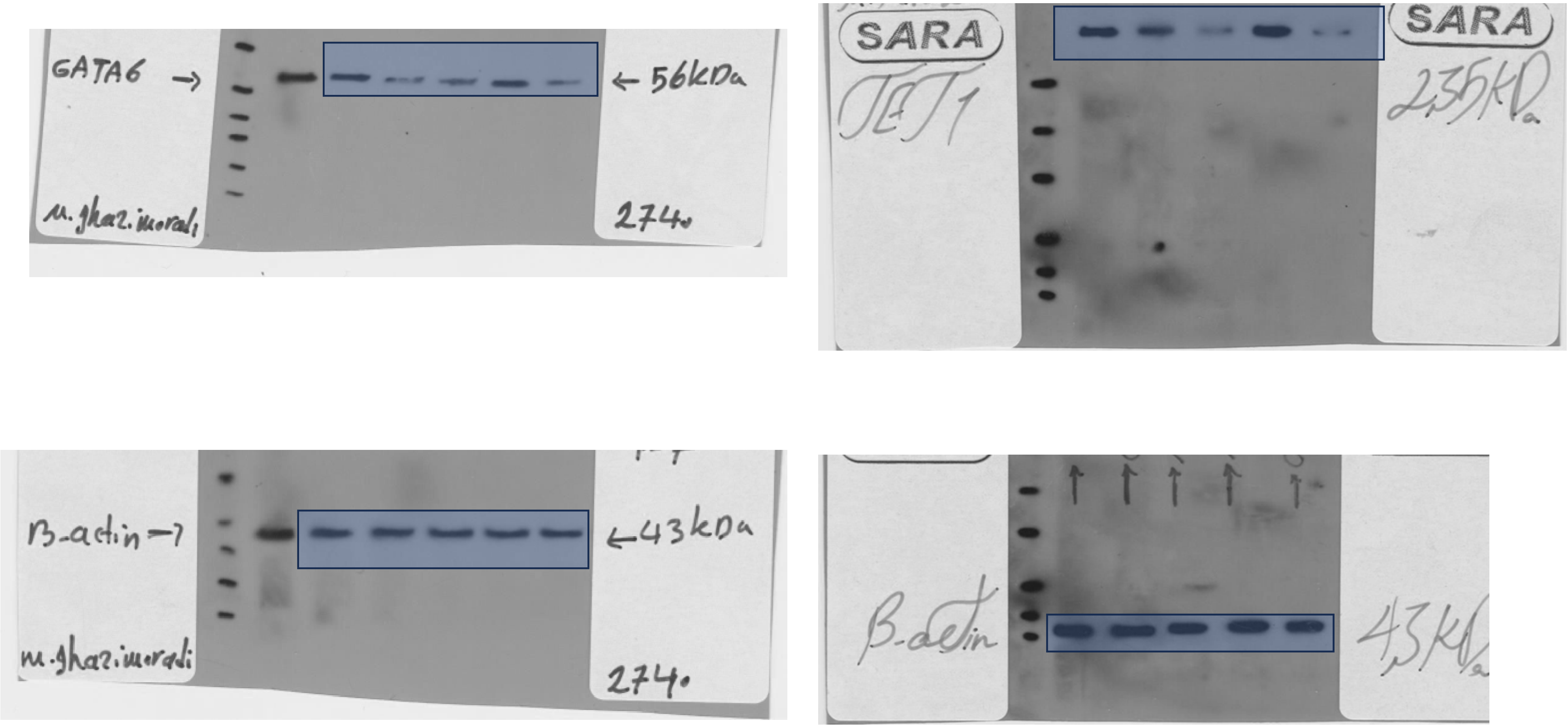

Supplementary Fig. 1. Western blots raw data. The full-length blots of GATA6, TET1, and β-actin.

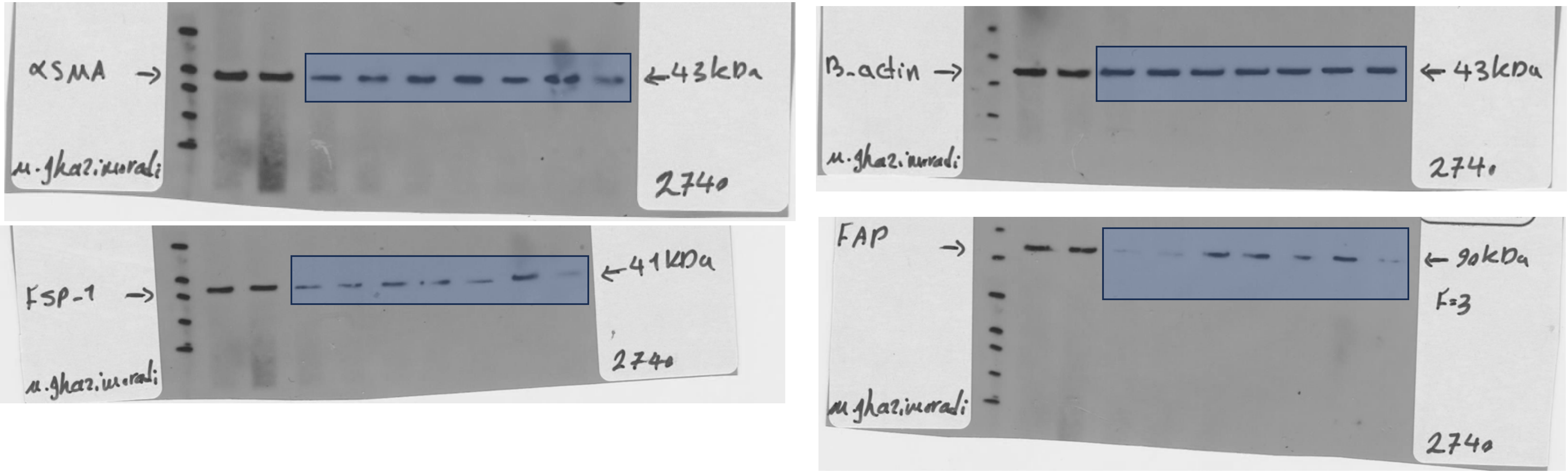

Supplementary Fig. 2. Western blots raw data. The full-length blots of αSMA, FSP, FAP, and β-actin.

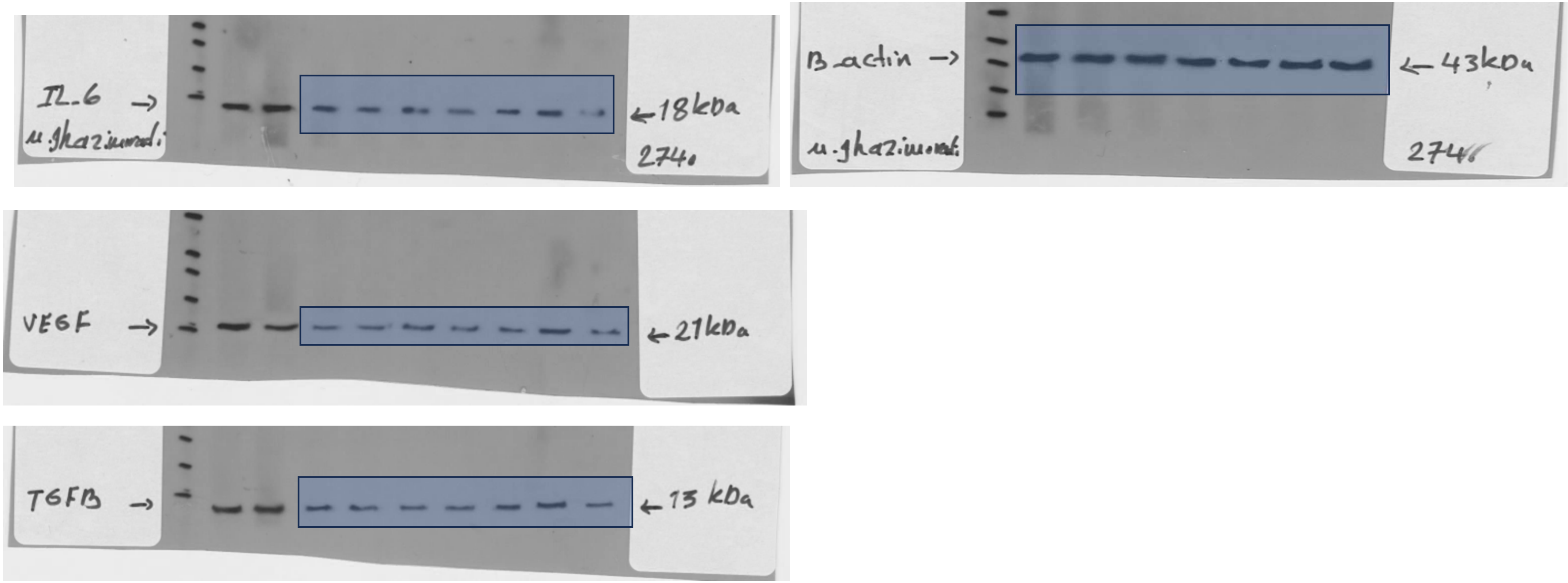

Supplementary Fig. 3. Western blots raw data. The full-length blots of IL6, VEGF, TGFβ, and β-actin.

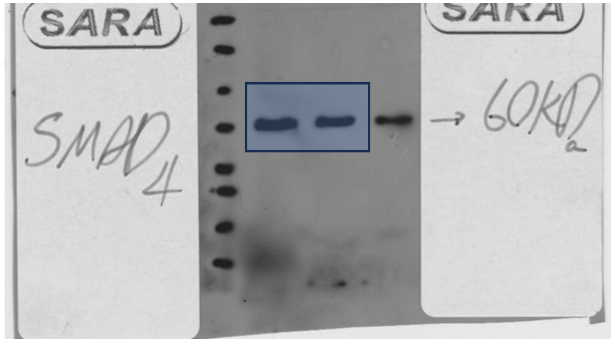

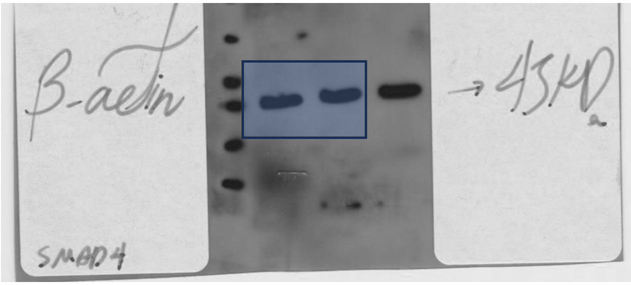

Supplementary Fig. 4. Western blots raw data. The full-length blots of SMAD4 and and β-actin.
